# Palynological Origin, Phenolic Content, and Antioxidant Properties of Honeybee-Collected Pollen from Bahia, Brazil

**DOI:** 10.3390/molecules17021652

**Published:** 2012-02-07

**Authors:** Kristerson R. L. Freire, Antonio C. S. Lins, Marcos C. Dórea, Francisco A. R. Santos, Celso A. Camara, Tania M. S. Silva

**Affiliations:** 1 Laboratório de Tecnologia Farmaceutica, Universidade Federal da Paraiba, CEP 58051-970, Paraiba, Brazil; 2 Departamento de Ciencias Biologicas, Universidade Estadual de Feira de Santana, CEP 44036-900, Bahia, Brazil; 3 Departamento de Ciencias Moleculares, Universidade Federal Rural de Pernambuco, CEP 52171-900, Pernambuco, Brazil

**Keywords:** bee pollen, antioxidant, palynological analysis, flavonoids

## Abstract

The aim of this study was to determine the palynological origin, phenolic and flavonoid content, and antioxidant properties of twenty-five samples of bee pollen harvested during a nine-month period (February–November) from the Canavieiras municipality (northeastern Brazil). Of the 25 samples analyzed, only two (February 01 and 02) were heterofloral. The predominant pollens in the samples analyzed during that month were: *Cecropia*, *Eucalyptus*, *Elaeis*, *Mimosa pudica*, *Eupatorium*, and *Scoparia*. Ethyl acetate fractions were analyzed by HPLC-DAD. The flavonoids isoquercetin, myricetin, tricetin, quercetin, luteolin, selagin, kaempferol, and isorhamnetin were detected. The flavonoid present in all 22 samples was isolated and identified as isorhamnetin 3-*O*-β-neohesperidoside. The total phenolic contents determined using the Folin-Ciocalteu reagent ranged from 41.5 to 213.2 mg GAE/g. Antioxidant activities based on the 1,1-diphenyl-2-picryl hydrazyl (DPPH), 2,2-azinobis 3-ethylbenzothiozoline-6-sulfonic acid (ABTS), and Fe^2+^ ion chelating activity assays were observed for all extracts, and correlated with the total phenolic content.

## 1. Introduction

Pollen is an interesting subject for apiculture, as it reveals the plants used in bee pasture. Bees harvest loads containing pollen grains, transporting them to hives and storing them in cells a good distance from the honeycombs. The pollen is used for feeding, especially of the larvae. The pollen grains are also good indicators of the botanical and geographical origin of bee products [1,2,3]. Bee pollen production is a recent activity in Brazil, having begun in the late 1980s. However, the country has the potential of becoming one of the world’s large producers of high quality pollen, particularly because of the great diversity in tropical flora and the resistance of the Brazilian *Apis mellifera* bee varieties. This biodiversity may allow the bees to collect pollen year-round. The municipality of Canavieiras (15°41′ S, 38°57′ W), located in the Litoral Sul economic microregion of the state of Bahia, is characterized by ample mangrove areas, but it also has large tracts of forest, restinga, and dune vegetation with an emphasis on palms (Arecaceae). Each of these ecosystems has rich flora with polliniferous and nectariferous plant species, which are important for apiculture. The quantity and quality of the bee pollen produced in this municipality has attracted increased attention to Brazilian apiculture [4]. This region produces over a ton of pollen per month.

Bee pollen has been used for many years in traditional medicine, supplementary nutrition, and in alternative diets, primarily due to its nutritional properties and health benefits. Honeybee-collected pollen is an apicultural product composed of nutritionally valuable substances and containing considerable amounts of polyphenolic compounds, primarily flavonoids, which may act as potent antioxidants [5]. 

In our continuing search for bee-pollen products [6,7,8], we have studied the palynological origin, phenolic content, flavonoid content, and antioxidant properties of twenty-five samples of bee pollen harvested from the Canavieiras municipality (northeastern Brazil) during a nine-month period (February–November).

## 2. Results and Discussion

### 2.1. Palynological and Flavonoid Analysis

Table 1 show the samples with Palynoteca UEFS registry numbers, the pollen types, and the flavonoids identified by HPLC-DAD (Figure 2). In the 25 samples, 11 different pollen types were identified. Approximately 50% of the identified taxons appear as predominant pollen (>45%), 16–45% appear as secondary pollen, and 3–15% appear as important minor pollen. Of the 25 samples, only two (Feb 01 and 02) are heterofloral. In the monofloral samples, those with a pollen type frequency greater than 45%, the predominant pollen types were: *Cecropia*, *Eucalyptus*, *Elaeis*, *Mimosa pudica*, *Eupatorium*, and *Scoparia*. Of the ten flavonoid standards used, only two (taxifolin and naringenin) do not appear in the analyzed pollen. The flavonoids isoquercetin, quercetin and isorhamnetin were identified in 80% of the samples. This suggests that these flavonoids are not specific to the plant species. The Mar 01–03 samples showed the presence of eight flavonoids that are monofloral from *Eucalyptus* (Table 1). The flavonoids myricetin, tricetin, and luteolin present in this pollen have been identified in *Eucalyptus globulus* Labill pollen [9]. The flavonoid selagin is common to these three samples as well. There are no reports of the isolation of the flavonoid selagin in *Eucalyptus* species. A substantial proportion of the flavonoids found in the *Eucalyptus* genus are aglycones. 

**Table 1:** Main pollen types present in samples collected from beehives in the municipality of Canavieiras (northeast Brazil) and the distribution of flavonoids in EtOAc fractions by HPLC-DAD.

	Palynoteca registry (PUEFS)	Pollen types		Flavonoids identified *							
			%	Isoq	Myri	Tri	Quer	Lut	Sel	Kae	Isor
1-Feb	528	Scrophulariaceae 2	35.85	X			X			X	X
		*Eupatorium*	16.82								
2-Feb	529	*Ricinus*	43.70	X			X				
		*M. arenosa*	26.73								
3-Feb	530	*Cecropia*	64.23	X		X	X			X	
		*Eupatorium*	12.19								
1-Mar	531	*Eucalyptus*	71.73	X	X	X	X	X	X	X	X
		*Cecropia*	21.80								
2-Mar	532	*Eucalyptus*	66.80	X	X	X	X	X	X	X	X
		*Cecropia*	24.60								
3-Mar	533	*Eucalyptus*	50.47	X	X	X	X	X	X	X	X
		*Cecropia*	32.33								
4-Mar	534	*Elaeis*	84.00	X			X	X			X
		Melast./Crombretaceae	10.80								
1-Apr	535	*M. pudica*	74.60	X			X			X	X
		*Elaeis*	16.16								
2-Apr	536	*Elaeis*	75.53	X			X				X
		*Eupatorium*	15.33								
May	537	*M. pudica*	60.20	X			X				X
		*Elaeis*	30.60								
1-Jun	540	*Eupatorium*	77.73	X			X			X	
		*Elaeis*	13.00								
2-Jun	912	*M. pudica*	77.07				X				
		Non-identified	8.52								
3-Jun	913	*Elaeis*	73.46								X
		*Elephantopus*	12.89								
1-Jul	542	*M. pudica*	97.13	X			X	X			X
		*Elaeis*	1.40								
2-Jul	545	*Cecropia*	65.40	X	X		X				X
		*Elaeis*	25.87								
3-Jul	546	*M. pudica*	93.87	X						X	
		*Eupatorium*	3.00								
1-Aug	547	*M. pudica*	77.53	X	X	X	X				X
		*Elaeis*	12.27								
2-Aug	549	*Elaeis*	60.73	X	X	X	X	X			X
		*Cecropia*	22.93								
3-Aug	550	*M. pudica*	76.07	X						X	X
		*Cecropia*	14.60								
Sep	551	*Eucalyptus*	36.07	X	X					X	X
		*Elaeis*	32.87								
1-Oct	552	*Elaeis*	48.63	X			X				X
		*Cecropia*	32.82								
2-Oct	553	*M. pudica*	74.80		X	X	X			X	X
		*Cecropia*	15.87								
3-Oct	554	*Elaeis*	99.33								X
		*Elephantopus*	0.53								
1-Nov	555	*Scoparia*	98.53		X						
		*Eucalyptus*	0.67								
2-Nov	556	*Eucalyptus*	57.80	X	X		X			X	X
		*Cecropia*	34.53								

* Isoq = isoquercetin; Myri= myricetin; Tri = tricetin; Quer = quercetin; Lut = luteolin; Sel = selagin; Kae = kaempferol; Isor = isorhamnetin.

Nine of the flavonoids used as standards in this work, except selagin, were isolated or previously identified in species of the genus *Eucalyptus*. Conde *et al.* (1997) [10] identified several flavonoids in the bark, aerial parts, and leaves of *E. globulus*, *E. camaldulensis* and *E. rudis*. The flavonoids isoquercetin, quercetin and myricetin were isolated from the leaves of *E. citriodora* [11]. Quercetin, myricetin, tricetin, luteolin, isorhamnetin, and kaempferol were found in *Eucalyptus* honey, reinforcing these flavonoids as chemical markers for honey from the monofloral species of this genus [12]. The monofloral pollen from *Elaeis*, *Mimosa*, *Cecropia*, *Mimosa pudica*, and *Eupatorium* appear as the majority in the months from February to October, representing plants commonly found in the area of collection that are in bloom during much of the year. The *M. pudica* pollen was found in large quantity in seven samples (April 01, May, June 02, July 01, August 01, August 03, October 02), corresponding to six consecutive months. The flavonoids isoquercetin, quercetin, and isorhamnetin were the most commonly found and were identified in six samples. The next most common flavonoids were kaempferol (four samples), myricetin and tricetin (two samples), and luteolin (one sample) (Table 1). The absence or presence of a flavonoid in the same vegetal species during the year may be associated with the need to develop certain bioactivities at certain times depending on climate, humidity, *etc.* The July 01 sample contains the pollen type *Mimosa pudica* as 97.13% of the total grains. The flavonoids identified were luteolin, isoquercetin, quercetin and isorhamnetin, the last three of which are the most common in samples containing a majority of the pollen type *M. pudica*. Of the flavonoids present in *M. pudica*, only myricetin was previously isolated from the aerial parts of *M. pudica* in Colombia [13]. The pollen type *Elaeis* was monofloral in six samples (March 04, April 02, June 03, August 02, September, October 03) corresponding to six months beginning in April and blooming every two months until October. The flavonoid isorhamnetin was identified in all of these samples, followed by isoquercetin (four samples), luteolin (two samples), and myricetin and tricetin (one sample). Isorhamnetin is commonly found in bee pollen samples [7]. The November 01 sample is monofloral from *Scoparia* (98.53%), and the flavonoid identified in the sample was myricetin. There are no reports of the isolation of this flavonoid in the *Scoparia* genus, but reports indicate the presence of other flavonoids such as apigenin and luteolin in *S. dulcis* [14]. The pollen type *Cecropia* was predominant in February 03 and July 02. The first identified flavonoids were tricetin and kaempferol, and the last were myricetin and isorhamnetin. Isoquercetin and quercetin were found in both samples. Traces of the pollen type *Eupatorium* can also be found in some samples. *Eupatorium* is monofloral in the June 01 sample with 77.73% of the total grains. The flavonoids identified were isorhamnetin, quercetin and kaempferol. Kaempferol was isolated from the flowers of *Eupatorium betonicaeforme* [15], luteolin from the flowers of *Eupatorium odoratum* [16], and isoquercetin from the aerial parts of *Eupatorium ballotaefolium* [17]. The *Ricinus* pollen was secondary pollen (43.70%) in the February 02 sample. Only the flavonoids quercetin and isoquercetin were identified. Isoquercetin [18], quercetin and kaempferol [19] were previously identified in the species *Ricinus communis* L.

**Figure 1:**
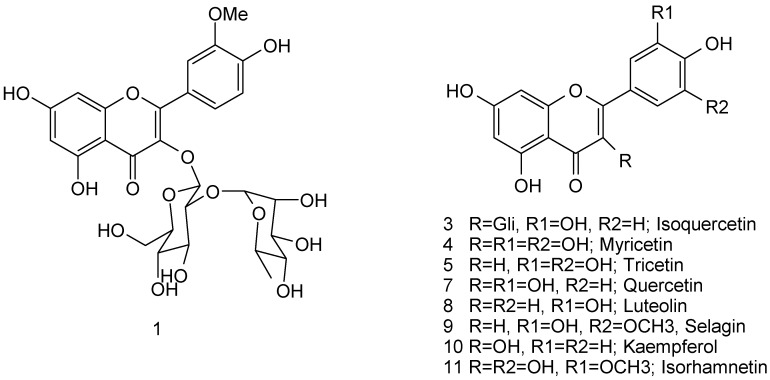
Flavonoids identified in the pollen.

NMR spectra were used to identify the structures of the flavonoids isolated from pollen as myricetin (**4**), quercetin (**7**), tricetin (**5**), and 3-*O*-β-neohesperidoside (**1**) (Figure 1). Their spectral data, particularly ^13^C-NMR, were in close agreement with the literature values for myricetin, quercetin, tricetin [20], and 3-*O*-β-neohesperidoside [21]. The flavonoid 3-*O*-β-neohesperidoside was previously isolated from *Typha angustifolia* pollen [22].

### 2.2. Total Phenolic Content, Antioxidant Activity, and Correlations between Total Phenolic and Metal Chelating Activity and Antiradical Activity

**Figure 2:**
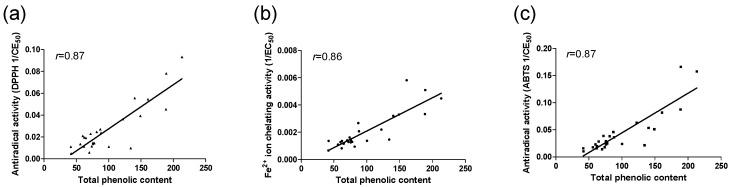
Correlation between the total phenolic content and the antiradical activity DPPH (**a**), between the total phenolic content and the metal chelating activity (**b**), and between the total phenolic content and antiradical activityABTS (**c**).

The total phenolic content for the EtOAc fractions ranged from 41.50 ± 0.24 to 213.22 ± 1.10 mg EAG/g (Table 2). The March samples possessed the highest levels: Mar 02 (213.22 ± 1.09 mg EAG/g), Mar 01 (188.92 ± 2.03 mg EAG/g), Mar 03 (160.60 ± 1.11 mg EAG/g), followed by Feb 02 (188.56 ± 1.76 mg EAG/g), Jun 03 (149.02 ± 0.83 mg EAG/g), Jun 01 (140.16 ± 0.62 mg EAG/g), and Nov 01 (134.10 ± 1.25 mg EAG/g). The samples corresponding to the month of March also had the largest number of flavonoids (Table 1). The pollen samples analyzed in the present work were shown to have considerable antioxidant activity and a great diversity of flavonoids (Table 1 and Table 2). From the chemical composition of the 25 samples, antioxidant activity was expected because literature reports detail antioxidant activity for all of the flavonoids [23,24]. Results shown in Table 2 are consistent with this expectation. It is interesting to observe the correlation between the phenolic content and the antioxidant activity among the pollen extracts because phenolic compounds contribute directly to antioxidant activity [25]. In this study, there was a distinct correlation between the studied parameters (total phenolic content, metal chelating activity, and antiradical activity) in the EtOAc fractions of the pollen. The relationships between the total phenolic content and the antiradical activity DPPH (1/EC_50_), metal chelating activity, and antiradical activity ABTS (1/EC_50_) are shown in Figure 2(a–c), respectively. The Pearson correlation coefficients (*r*) of these plots were approximately 0.8 for all EtOAc fractions of the pollen.

**Table 2:** Total phenolic content and antioxidant activity of pollen samples.

Sample	Total phenolic content (mg GAE/g) ^a^	DPPH (EC_50_) ^a,^^b^	ABTS (EC_50_) ^a,b^	Fe^2+ ^ion chelating activity ^a,b^
Feb 01	88.1 ± 0.5	42.3 ± 1,1	27.7 ± 0.4	483.6 ± 7.6
Feb 02	188.6 ± 1.7	22.0 ± 0.0	11.4 ± 0.1	299.7 ± 6.3
Feb 03	122.1 ± 1.3	27.5 ± 0.0	15.8 ± 0.5	455.5 ± 21.4
Mar 01	188.9 ± 2.0	12.8 ± 0.1	6.0 ± 0.1	196.2 ± 4.3
Mar 02	213.2 ± 1.1	10.7 ± 0.0	6.4 ± 0.1	223.0 ± 4.8
Mar 03	160.6 ± 1.1	18.3 ± 0.5	12.2 ± 0.2	171.9 ± 5.9
Mar 04	75.7 ± 1.6	73.2 ± 0.7	43.5 ± 0.8	706.2 ± 13.3
Apr 01	77.6 ± 1.3	69.7 ± 0.8	40.4 ± 1.1	772.5 ± 3.5
Apr 02	69.9 ± 0.3	17.9 ± 0.2	71.2 ± 1.7	712.1 ± 14.4
May	61.8 ± 2.0	88.7 ± 1.6	63.4 ± 0.9	1214.1 ± 40.3
Jun 01	140.1 ± 0.6	18.0 ± 0.0	18.8 ± 0.7	312.4 ± 9.3
Jun 02	75.6 ± 1.3	68.0 ± 0.4	34.4 ± 1.8	795.9 ± 21.6
Jun 03	149.0 ± 0.8	25.5 ± 10.8	19.6 ± 0.3	303.3 ± 6.1
Jul 01	59.9 ± 0.8	47.8 ± 0.3	43.7 ± 0.4	781.8 ± 3.9
Jul 02	62.2 ± 0.5	51.1 ± 0.5	51.1 ± 3.2	737.8 ± 11.4
Jul 03	81.4 ± 2.8	40.6 ± 0.3	26.2 ± 2.0	1063.6 ± 7.9
Aug 01	41.5 ± 0.2	90.1 ± 0.4	64.1 ± 3.5	1507.0 ± 40.8
Aug 02	55.9 ± 0.4	74.6 ± 0.1	57.6 ± 1.2	909.9± 9.6
Aug 03	73.8 ± 0.3	87.1 ± 0.5	56.3 ± 2.5	6196 ± 12.9
Sep	64.8 ± 1.7	52.2 ± 0.0	35.5 ± 1.8	861.6 ± 7.8
Oct 01	100.2 ± 1.1	90.4 ± 0.1	41.9 ± 1.8	727.3 ± 7.5
Oct 02	71.9 ± 1.3	43.8 ± 0.0	25.8 ± 1.2	777.1 ± 19.4
Oct 03	41.9 ± 1.1	209.1 ± 0.5	97.2 ± 3.8	734.4 ± 8.8
Nov 01	134.1 ± 1.2	102.1 ± 0.5	46.7 ± 0.2	684.24 ± 5.3
Nov 02	86.9 ± 1.1	37.1 ± 0.1	21.8 ± 0.3	373.4 ± 11.7
Ascorbic acid		2.5 ± 0.0		
Trolox			2.7 ± 0.0	
EDTA				5.1 ± 0.1

^a^ Mean value± standard deviation: n = 3; ^b^ Concentration of antioxidant required to reduce the original amount of the radicals by 50%.

## 3. Experimental

### 3.1. Materials and Methods

#### 3.1.1. Reagents and Standards

Taxifolin (**2**), isoquercetin (Isoq, **3**), myricetin (Myri, **4**), tricetin (Tri, **5**), naringenin (**6**), quercetin (Quer, **7**), luteolin (Lut, **8**), selagin (Sel, **9**), kaempferol (Kae, **10**), and isorhamnetin (Isor, **11**), were previously isolated and identified from pollen loads [6,7,8]. Folin-Ciocalteu’s phenol reagent, 1,1-diphenyl-2-picryl hydrazyl (DPPH), potassium persulfate, 6-hydroxy-2,5,7,8-tetramethylchroman-2-carboxylic acid (Trolox), beta-carotene, linoleic acid and ferrozine were obtained from Sigma-Aldrich (Sternheim, Germany), and butylated hydroxytoluene (BHT) was supplied by Acros Organics (New Jersey, USA). Gallic acid and 2,2-azinobis 3-ethylbenzothiozoline-6-sulfonic acid (ABTS), were purchased from Fluka Chemie (Buchs, Switzerland). Ascorbic acid, ethylenediaminetetraacetic acid (EDTA) and HPLC grade formic acid were purchased from Vetec (Rio de Janeiro, Brazil). Methanol (HPLC grade) was from Tedia (Rio de Janeiro, Brazil).

#### 3.1.2. General Procedures

Melting points were determined on a Kofler hot stage and are uncorrected. Silica gel 60 F254 (Merck) was used for the TLC plates. The absorbance of the solutions in the antiradical tests (DPPH and ABTS), the metal chelating activity on the ferrous ions (Fe^2+^), and the total phenolic content were determined on a Varian Cary® 50 UV-Vis Spectrophotometer. Sephadex® LH-20 was obtained commercially from Aldrich. All solvents used were of commercial and HPLC grade. Nuclear magnetic resonance (NMR) spectra were recorded on a JEOL FX-400 spectrometer operated at 400 MHz for ^1^H-NMR and at 100 MHz for ^13^C-NMR. The spectra were obtained on DMSO containing TMS as an internal standard.

### 3.2. Pollen Samples and Their Classification

Twenty-five pollen samples: February (Feb) 01–03, March (Mar) 01–03, April (Apr) 01 and 02, May, June (Jun) 01–03, July (Jul) 01–03, August (Aug) 01–03, September (Sep), October (Oct) 01–03, November (Nov) 01 and 02 (Table 1) were collected from apiarists in the Cooperative of Canavieiras City, Bahia, Brazil. The number of samples collected from the same geographic region each month was determined by the availability of pollen in the apiary from February to November 2003. Loads containing 2.0 g pollen were hydrated in water to free the pollen grains from the load mass. After being completely homogenized, the pollen grains were dehydrated in glacial acetic acid and the pollen sediments were prepared for palynological analysis using the acetolysis method [26]. Each sample was mounted on five slides with glycerin jelly (stained with safranin). The pollen grains were counted (at least 500 per slide) to establish the frequency of the pollen types. The pollen types were identified according to the recommendations of Santos [27] and by comparison with slides from the palynoteca of the Plant Micromorphology Laboratory (Universidade Estadual de Feira de Santana, Brazil). 

### 3.3. High Performance Liquid Chromatography-diode-array Detection (HPLC-DAD)

Each sample of bee pollen weighing from 4.5 to 43.9 g was subjected to successive extractions with EtOH in an ultrasonic bath. The EtOH extract was suspended in MeOH-H_2_O (1:1) and partitioned three times with *n*-hexane and then with EtOAc. The extract solutions obtained were concentrated on a rotary evaporator under reduced pressure, affording *n*-hexane and EtOAc extracts, respectively. The EtOAc fractions were dissolved in methanol (10 mg/mL) and filtered through a 0.45 μm nylon membrane for HPLC-DAD analysis. Dried extracts were stored in a refrigerator at 4 °C until further analysis. Chromatographic analyses were carried out using a Shimadzu HPLC SCL-10Avp equipped with a SPD-M10Avp photodiode array detector with a reversed-phase column LiChrosorb RP-18 column (250 mm × 4 mm × 5 μm, Merck) using a water–formic acid mixture (99:1, v/v solvent A) and MeOH (solvent B) as the mobile phase: 0–3 min 40% B, 5–15 min 45% B, 17–25 min 50% B, 27–43 min 55% B, 45 min 40% B, 1.0 mL/min at 360 nm. The pollen was screened for the presence of the following available standard flavonoids: taxifolin (**2**), isoquercetin (**3**), myricetin (**4**), tricetin (**5**), naringenin (**6**), quercetin (**7**), luteolin (**8**), selagin (**9**), kaempferol (**10**), and isorhamnetin (**11**). Retention times and the UV-Vis spectra of the flavonoids in the pollen (Figure 1) were compared with those of aglycone standards.

### 3.4. Extraction and Separation

Two samples were analyzed for the separation of flavonoids. The Feb–Mar pollen sample (162.3 g) was extracted with ethanol in an ultrasonic bath, and the extract was filtered and concentrated under reduced pressure. The crude residue (83.5 g) was dissolved in MeOH-H_2_O (7:3) and extracted with hexane, followed by EtOAc. These solvents were evaporated to dryness. An aliquot of the EtOAc fraction (400.0 mg) was successively submitted to column chromatography using a Sephadex LH-20 column with methanol as the eluent. The collected flavonoid fractions were analyzed by thin layer chromatography, and the spots were made visible by spraying them with a 1% diphenylboryloxyethylamine (NP) solution in methanol and observing them under UV light at 366 nm. From this fraction the flavonoids **8** (12.8 mg), **7** (5.0 mg), and a mixture of **8**, **7** and **6** (5.0 mg) were isolated. The compound **6** was tested for anti-allergic effects in ovalbumin-sensitized mice [28]. 

**Figure 3:**
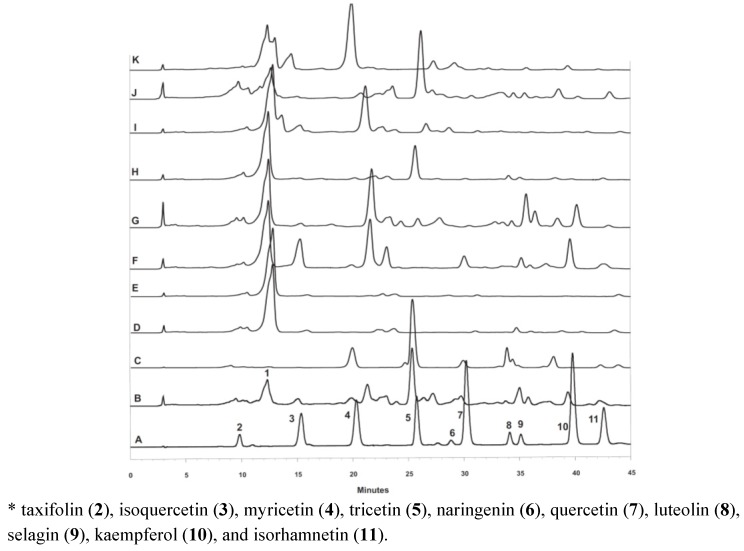
Chromatograms of flavonoids standards (A)* and the February 03 (B), March 04 (C), April 01 (D), May (E), June 01 (F), July 03 (G), August 02 (H), September (I), October 02 (J), November 02 (K) samples.

The Apr–May–Jul pollen sample corresponds to the combination of some EtOAc fractions (1.95 g) that demonstrated the presence of an intense and wide peak during the HPLC-DAD analyses. This compound appeared in 22 samples with a retention time of approximately 12.5 minutes at 360 nm (Figure 3) and was different from all of the flavonoid standard peaks. The EtOAc fractions were fractionated according to the procedure described above and yielded the flavonoid **1** (14.8 mg), which was identified as 3-*O*-β-neohesperidoside. Amorphous yellow solid; mp 185.5–187.5 °C; ^1^H-NMR δ: 7.9 (d; *J *= 7.2 Hz, H-2'), 7.5 (d; *J *= 8.0, H-6'), 6.9 (s, H-5'), 6.4 (s, H-8), 6.2 (s, H-6), 5.8 (d; 7.2 Hz, H-1"), 5.4 (sl, H-1'"), 4.6 (sl, H-4'"), 3.7 (sl, H-3'"), 3.8 (s, OCH_3_-3'), 3.7 (sl, H-5'"), 3.5 (sl, 2", H-2'"), 0.63 (sl, 6'"). ^13^C-NMR δ: 177.3 (C-4), 164.2 (C-7), 161.2 (C-5), 156.4 (C-9), 156.1 (C-2), 149.4 (C-3'), 146.8 (C-4'), 132.6 (C-3), 121.1 (C-1'), 104.0 (C-10), 121.7 (C-6'), 115.2 (C-5'), 113.5 (C-2'), 100.8 (C-1'''), 98.8 (C-1′′), 98.3 (C-6), 93.8 (C-8), 77.7 (C-2''), 77.4 (C-3''), 77.1 (C-5''), 71.7 (C-4'''), 70.6 (C-4''), 70.6 (C-2'''), 70.1 (C-3'''), 68.3 (C-5'''), 60.5 (C-6''), 17.0 (C-6'''), 55.7 (OCH_3_). 

### 3.5. Determination of Total Phenolic Content

The total phenolic content of the samples was determined using the Folin-Ciocalteu reagent according to the method of Slinkard and Singleton [29] modified by the use of gallic acid as a standard phenolic compound. Appropriate amounts of EtOAc fractions (500 μL, 50 µg/mL) were diluted in a volumetric flask with distilled water (3 mL). The Folin-Ciocalteu reagent (100 µL) was added, and the contents of the flask were thoroughly mixed. After 3 min, Na_2_CO_3_ (15%, 300 µL) was added, and the mixture was completed with distilled water (5 mL) and allowed to stand for 2 h in an ultrasonic bath. The absorbance was measured at 760 nm in a spectrophotometer. The total amount of phenolic compounds was determined in micrograms of gallic acid equivalents, using the equation obtained from the standard gallic acid graph.

### 3.6. DPPH^•^ Radical Scavenging Assay

The free radical scavenger activity was determined using the DPPH assay as previously described [7]. The antiradical activity was evaluated using a dilution series to obtain five concentrations. This involved mixing the DPPH solution (60 µM in ethanol) with an appropriate amount of the EtOAc fractions. After 30 min, quantification of the remaining DPPH radicals was recorded using absorption at 517 nm. The percentage of inhibition was given by the following formula: Percent inhibition (%) = [(A0 − A1) / A0] × 100 in which A0 is the absorbance of the control and A1 is the absorbance in the presence of the sample and standards. 

### 3.7. ABTS^+•^ Radical Cation Decolorization Assay

The radical cation decolorization assay was based on a method previously reported in the literature [30]. ABTS was dissolved in water to yield a final concentration of 7 mM. The ABTS radical cation (ABTS^+•^) was produced by reacting an ABTS stock solution with 2.45 mM potassium persulfate (final concentration) and allowing the mixture to stand in the dark at room temperature for 12–16 h before use. The ABTS^+•^ solution was diluted with ethanol to give an absorbance of 0.700 ± 0.025 at 734 nm prior to its use. Then, the appropriate amounts of the ABTS^+•^ solution were added to sample solutions in ethanol (5 mL) of the EtOAc fractions. After 10 min, the percentage inhibition of absorbance at 734 nm was calculated for each concentration relative to the blank absorbance (ethanol). The capability of scavenging the ABTS^+•^ radical was calculated using the following equation: ABTS^+•^ scavenging effect (%) = [(A_0_ − A_1_ / A_0_) × 100] in which A_0_ is the initial concentration of the ABTS^+•^ and A_1_ is the absorbance of the remaining concentration of ABTS^+•^ in the presence of the sample.

### 3.8. Metal Chelating Activity on Ferrous Ions (Fe^2+^)

The metal chelating activity was determined according to a method previously reported in the literature [31] with some modifications. Five concentrations of the EtOAc fractions were mixed with FeCl_2_ (2 mM, 75 μL) and ferrozine (5 mM, 300 μL). The solutions were diluted to 5 mL with H_2_O. Then, the mixtures were shaken vigorously and left to stand at room temperature for 10 min. After the mixtures reached equilibrium, the absorbance of the solutions was measured at a wavelength of 562 nm using a spectrophotometer. The chelating effect on the Fe^2+^ ions was calculated as the percentage (%) of inhibition of the ferrozine–Fe^2+^ complex formation determined as [(A_control_ − A_sample_)/A_control_] × 100 in which A_control_ is the absorbance of the ferrozine–Fe^2+^ complex alone, and A_sample_ is the absorbance of the test samples and the ferrozine–Fe^2+^ mixture. EDTA was used as the positive control. 

### 3.9. Statistical Analysis

All samples were analyzed in triplicate unless stated otherwise, and the results were expressed as the average ± the standard deviation. The ABTS, DPPH, and metal chelating activity on the ferrous ions (Fe^2+^) were tested at five different concentrations. All statistical analyses were performed using the Microsoft Excel software package (Microsoft Corp., Redmond, WA, USA). To determine whether the bioactive samples contributed to the antioxidant capacity, Pearson’s correlation coefficients were calculated.

## 4. Conclusions

With the results obtained by the HPLC-DAD analysis of samples of bee pollen (*Apis mellifera*) collected in the region of Canavieiras, Brazil, the determination of the groups of plants visited by the bees was possible. In addition, chromatographic profiles of the flavonoids present during the nine-month period could be determined. Some flavonoids such as isoquercetin, quercetin and isorhamnetin may be not specific to plant species, appearing in more than 90% of the samples. During the months from February to November, the bees visited three main genera of plants common to the region: *Mimosa* (Leguminosae), *Eucalyptus* (Myrtaceae) and *Elaeis* (Arecaceae). *Eucalyptus* is present in large reforestation areas; *Elaeis* is found in the coastal “restinga” and forests of Canavieiras; and *Mimosa* covers a very common group of plants in the Bahia region. Antioxidant activities based on the DPPH, ABTS, and chelating activity on the Fe^2+^ ions were observed for all fractions and correlated with the total phenolic content. In conclusion, this study reports, for the first time, the potential antioxidant composition of the most important monofloral pollens from Brazil and confirms that they contain relevant flavonoids. This research is the first step towards certification and quality control of pollen production in northeastern Brazil. More detailed and specific studies may be performed to fully assess this product that has become quite popular for its medicinal properties.
